# A new species of *Astragalus* (Fabaceae) from the Irano-Turanian biodiversity hotspot: an integrative approach

**DOI:** 10.1186/s40529-024-00448-6

**Published:** 2025-01-08

**Authors:** Zahra Karbalaei, Ali Bagheri, Ali Asghar Maassoumi, Twan Rutten, Frank R. Blattner

**Affiliations:** 1 Department of Plant and Animal Biology, Faculty of Biological Science and Technology, University of Isfahan, Isfahan, 81746-73441 Iran; 2 Botany Research Division, Research Institute of Forests and Rangelands, Agricultural Research, Education and Extension Organization (AREEO), Tehran, 13185–116 Iran; 3 Leibniz Institute of Plant Genetics and Crop Research (IPK), 06466 Gatersleben, Germany; 4 German Centre of Integrative Biodiversity Research (iDiv) Halle-Jena-Leipzig, 04103 Leipzig, Germany

**Keywords:** *Astragalus kuzehrashensis*, Endemic species, Genome size, Molecular phylogeny, Seed micromorphology, Species delimitation, Taxonomy

## Abstract

**Background:**

The genus *Astragalus* is the largest and one of the most diverse genera of flowering plants, particularly in the Northern Hemisphere, with a significant concentration of species in the Irano-Turanian region. Within this genus, section *Hymenostegis* is notable for its complexity and high levels of endemism, especially in northwestern Iran. During recent field explorations in West Azarbaijan province, a distinct population of *Astragalus* was identified, differing from known species within section *Hymenostegis*. This study aimes to describe and analyze this new species and clarify its relationship with closely related taxa using an integrative approach.

**Results:**

*Astragalus kuzehrashensis* sp. nov. is described following detailed morphological comparisons with its closest relatives, *A. chehreganii* and *A. hakkianus*. The new species is distinguished by unique stipule characteristics, leaflet shape, inflorescence structure, and seed micromorphology. Molecular phylogenetic analysis using ITS and *ycf*1 sequences reveals that *A. kuzehrashensis* forms a clade with *A. chehreganii* and *A. hakkianus*, although it exhibits genetic differences. Genome size estimation confirms that *A. kuzehrashensis* is hexaploid (2*n* = 6*x* = 48), aligning it more closely with *A. hakkianus* than with the tetraploid *A. chehreganii*. The distinct morphological characters and genomic data support recognizing *A. kuzehrashensis* as a new species within section *Hymenostegis*.

**Conclusions:**

The identification of *A. kuzehrashensis* underscores the importance of an integrative taxonomic approach, combining morphological, molecular, and cytogenetic data to resolve species boundaries within complex groups like *Astragalus* section *Hymenostegis*. This study highlights the rich biodiversity of the Irano-Turanian floristic region, emphasizing the need for ongoing exploration and conservation efforts, particularly in areas of high endemism. Our findings contribute to a deeper understanding of the taxonomy and evolutionary relationships within the genus *Astragalus*.

**Supplementary Information:**

The online version contains supplementary material available at 10.1186/s40529-024-00448-6.

## Introduction

The genus *Astragalus* L. (Fabaceae) is one of the most diverse and widespread genera of flowering plants, particularly in the Northern Hemisphere. It is characterized by remarkable morphological diversity and high levels of endemism, especially in Southwestern Asia (Podlech and Zarre 2013; Maassoumi 2022). *Astragalus* is the largest genus of flowering plants globally, with over 3,000 species described worldwide, including more than 850 species found in Iran alone (Maassoumi 2020; Ghahremaninejad et al. 2022). The species within this genus are adapted to a wide range of ecological conditions, from cold to warm, arid, and semi-arid mountainous regions (Wojciechowski 2005). With around 255 sections, the genus displays considerable taxonomic complexity. Among its global distribution, Iran is recognized as one of the most important centers of diversity for this genus (Maassoumi 2022).

Within *Astragalus*, the spiny section *Hymenostegis* Bunge is particularly noteworthy due to its complexity and diversity. This section is one of the largest sections of the Old World *Astragalus*, predominantly occurring in Iran and Turkey (Bagheri et al. 2016). *Hymenostegis* includes 81 recognized species worldwide (Maassoumi 2020), nearly 75% of which are endemic to Iran, establishing Iran as the center of diversity for this taxon (Ghahremaninejad 1992; Bagheri et al. 2014; Ghahremaninejad et al. 2016). Its species are distinguished by specific morphological characters such as an inflated calyx, basifixed hairs, absence of black hairs, and conspicuous bracts (Zarre and Podlech 1996), highlighting the importance of hair characteristics in the taxonomy of *Astragalus* taxa (Zarre 2003; Ghahremaninejad 2004). Previous studies have provided significant insights into the taxonomy and phylogeny of *Hymenostegis*. Comprehensive molecular phylogenetic analyses revealed that *Hymenostegis* is not monophyletic in its current circumscription, as some species from neighboring sections group within *Hymenostegis* (Bagheri et al. 2017). Additionally, molecular studies highlighted the relatively young evolutionary age of the species within this section, generally less than 1 million years, resulting in polytomies in phylogenetic trees of this section. This is an indication for ongoing rapid species radiation, which is even more obvious in the considerable morphological variation observed among the *Hymenostegis* species (Bagheri et al. 2017). Beyond molecular data, studies on genome size and chromosome numbers have also contributed to our understanding of this section. Our previous work (Bagheri et al. 2022) provided genome size estimates for selected taxa belonging to this section and identified correlations between genome size, ploidy levels, and chromosome numbers in *Hymenostegis*. This shows that integrating cytological data with molecular and morphological analyses helps to identify polyploids and resolve taxonomic ambiguities within this group.

During field explorations conducted over the last decade in northwestern Iran, an unusual population of *Astragalus* was collected four times in four different years in the same vicinity. Initial morphological comparisons suggested that this population resembled *Astragalus chehreganii* Podlech & Zarre (Zarre and Podlech 1996) and *Astragalus hakkianus* Bagheri, Maassoumi & Rahimin. (Bagheri et al. 2013). However, further examination revealed significant differences, particularly in stipule size and texture, leaflet shape and indumentum, peduncle length and indumentum, inflorescence shape, and calyx indumentum. In addition to these analyses, we conducted genome size estimation and chromosome counting for the newly identified species. We also constructed a phylogenetic tree based on nuclear rDNA ITS and chloroplast *ycf*1 sequences for this unknown species and its close relatives, building upon our previous work (Bagheri et al. 2017).

Moreover, seed micromorphological studies using Scanning Electron Microscopy (SEM) provided valuable insights into the subtle morphological differences among the species in this complex, further aiding in their differentiation (Rashid et al. 2018; Zhao et al. 2023). Seed micromorphology has led to the discovery and confirmation of new taxa and has clarified relationships among closely related species within the genus *Astragalus* (Brullo et al. 2013; Bagheri et al. 2015; Kashyap et al. 2021).

The main aim of this study was to comprehensively analyze and describe the novel species and to employ an integrated approach, encompassing seed micromorphology, phylogenetic analysis, and genome size estimation, to clarify its differentiation from closely related species within the genus *Astragalus*.

## Materials and methods

### Study area

**Fig. 1:**
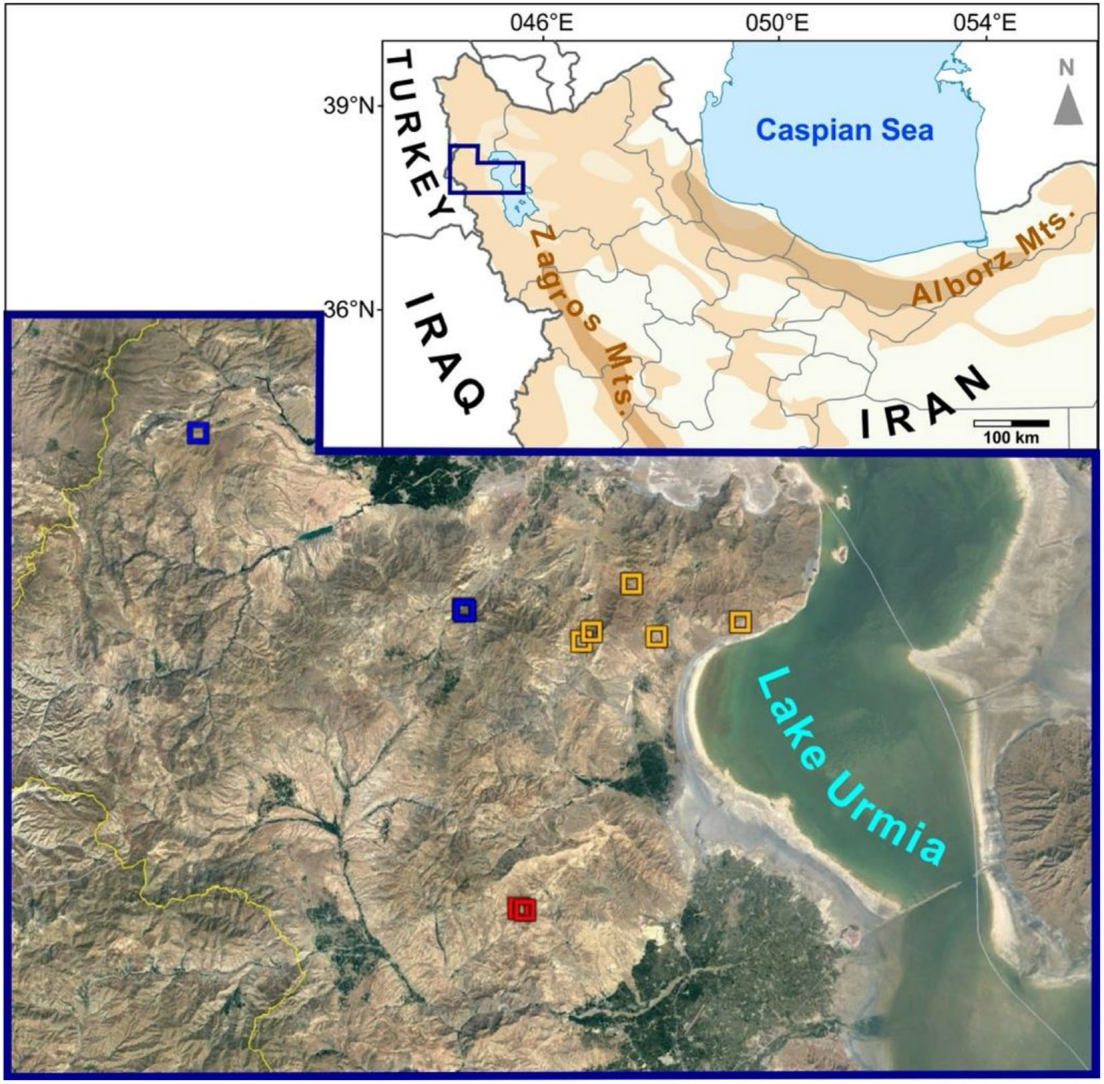
Study area in northwestern Iran. Sample sites of the three closely related species are indicated by colored squares, with *A. chehreganii* in orange, *A. hakkianus* in red, and *A. kuzehrashensis* sp. nov. in blue. The satellite photo of the northern reaches of Lake Urmia was taken from Google Earth

The plant specimens examined in this study were collected from the West Azarbaijan province, located in northwestern Iran, between Lake Urmia and the Turkish border. This region forms a significant part of the Irano-Anatolian biodiversity hotspot in Southwest Asia, where over 40% of the taxa are endemic to this area (Mittermeier et al. 2011). The study area, encompassing approximately 134,000 km², includes the sites where all three species under investigation (*A. chehreganii*, *A. hakkianus*, and the newly described species) were collected. The sampling sites form a roughly triangular shape within this region, as depicted in Fig. 1. This area includes elevational and microclimate differences. The geographical closeness and similar environmental conditions among the collection sites are critical for understanding the subtle morphological and genetic variations observed within this species group. The region is characterized by diverse topographical and climatic conditions, typical of the northern slopes of the Zagros Mountains (Noroozi et al. 2020). The vegetation is primarily composed of steppe and mountainous grasslands, providing a suitable habitat for many Irano-Turanian elements like *Astragalus* species, particularly those in the *Hymenostegis* section.

### Sampling

**Fig. 2:**
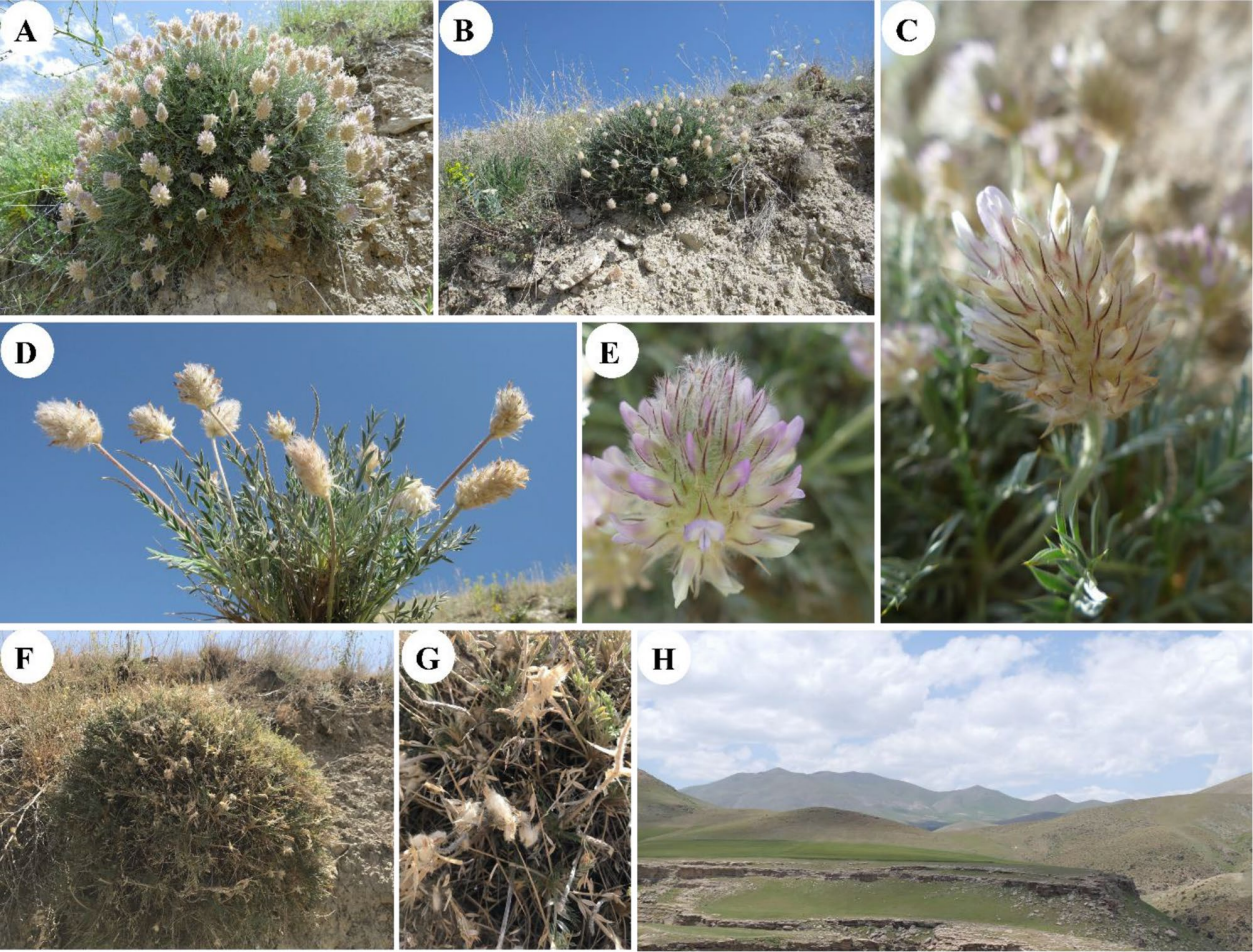
Morphological features and habitat of *Astragalus kuzehrashensis*. **A** and **B** Full view of the cushion growth form shrub in its natural habitat on a rocky slope; **C** Detail of inflorescence showing flower structure at anthesis; **D** Side view of an inflorescence, highlighting the arrangement of flowers; **E** Close-up of a single flower displaying the petal and calyx teeth; **F** and **G** Plant in the seed stage, showing dried inflorescences, seed pods, and dried flowers; **H** Landscape of the type locality near Kuzehrash, illustrating the typical habitat where *A. kuzehrashensis* is found

Over the past decade, sampling was conducted across four different years, with six separate visits to the study area. Fresh plant specimens were collected from June to July, during the peak of the vegetative stage, for herbarium sheet preparation, and leaves were gathered for further studies. Leaves were also preserved in silica gel to ensure rapid drying for subsequent genome size estimation or molecular systematic studies. Given the importance of seeds in our analysis of this species complex, additional sampling was conducted later in the growing season, specifically in August, to collect mature seeds. On average, 3 to 5 individuals from each species within this complex (*A. chehreganii*, *A. hakkianus*, and the newly identified species) were collected during each sampling event. These samples were subsequently used in various analyses, including morphological, micromorphological, cytological, and molecular studies. The exact locations of the sampling sites were recorded using GPS coordinates and altitude. Additionally, photographs of the plants in their natural habitats were taken, with images of the newly identified species presented in Fig. 2.

### Morphological studies

Initial assessments of the unknown specimens indicated that the diagnostic features necessary for precise identification were required within this specific species group, which includes *A. chehreganii* and *A. hakkianus*. Therefore, we focused our morphological analysis exclusively on this group and did not extend our analysis to other species within the section *Hymenostegis*. In this study, key morphological characters were carefully examined and documented to distinguish between *A. chehreganii*, *A. hakkianus*, and the newly identified species. The identification process relied on the use of identification keys provided in relevant taxonomic references (Podlech and Maassoumi 2001; Podlech and Zarre 2013; Maassoumi 2016). To facilitate the differentiation of these closely related species, a diagnostic table was constructed, highlighting the distinguishing morphological features within this species group (Table 1). This table includes comparative details of characters such as leaf hairs, stipule structure, inflorescence characteristics, and other diagnostic features consistently observed across multiple individuals. In addition to field observations, herbarium studies were conducted to further validate the morphological findings. The type specimens of *A. chehreganii* and *A. hakkianus* were carefully reviewed and compared with the newly collected specimens. These herbarium studies were performed in the herbaria MSB, TARI, and HUI, where the type materials of the relevant species are preserved. The integration of field observations, herbarium specimen comparisons, and identification keys provided a robust framework for accurate species identification within this complex.

### Seed micromorphology

For the seed micromorphological surveys, air-dried samples from this species complex were placed onto carbon adhesive discs, gold-coated using an Edwards S150B sputter coater (Edwards High Vacuum Inc., http://www.edwardsvacuum.com), and examined with a Zeiss Gemini300 scanning electron microscope (Carl Zeiss Microscopy GmbH, https://www.zeiss.de) at an acceleration voltage of 10 kV. The images were stored as TIFF files. Micrographs were captured at different magnifications (20×, 500×, 1000×, and 2500×) for three individuals of each species. The terminology used for describing seed sculpture was based on the relevant literature (Bojnansky and Fargasova 2007; Meyers et al. 2013).

### Phylogenetic analysis

**Fig. 3:**
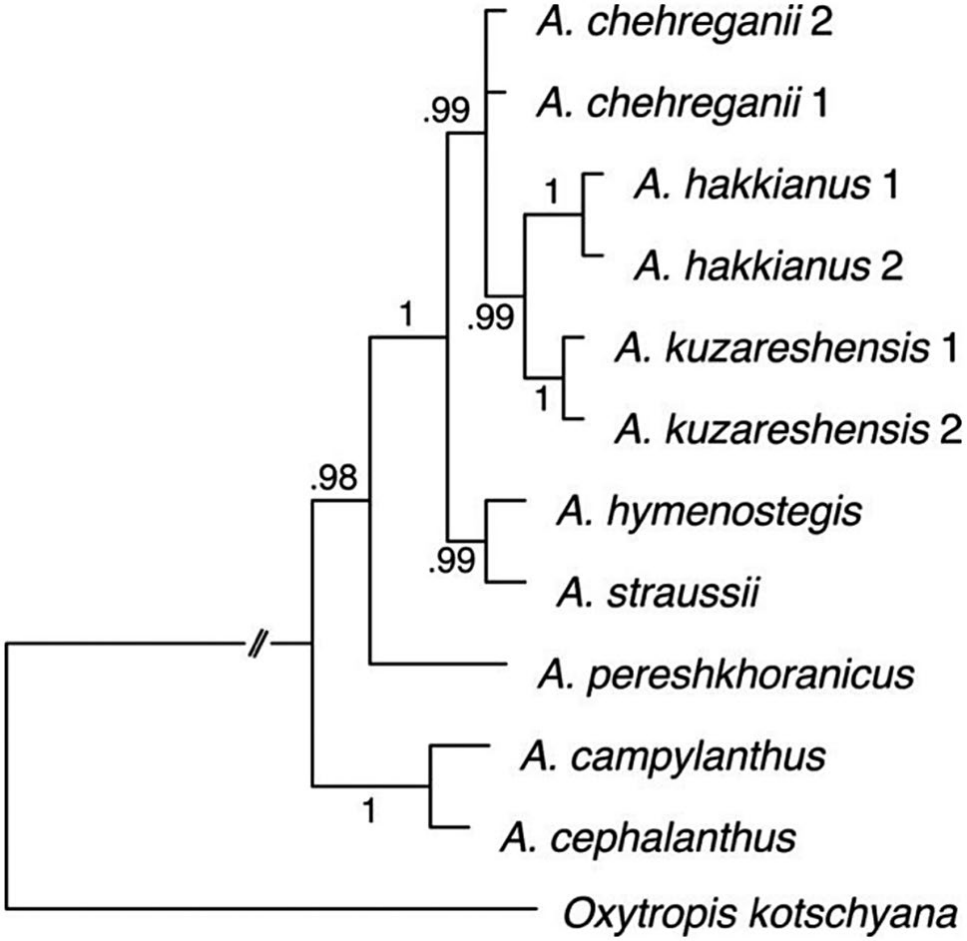
Bayesian phylogenetic tree based on combined ITS and *ycf*1 sequences, showing the genetic relationships among species within the studied complex, along with other species and the outgroup. Posterior probabilities (pp) are indicated along the branches

For the molecular phylogenetic analysis of this species group, we included nine individuals from section *Hymenostegis*, consisting of two individuals from each of the three species under study (*A. chehreganii*, *A. hakkianus*, and the newly identified taxon). Additionally, we included one individual from three other species of this section, namely *A. hymenostegis* Fisch. & C.A.Mey., *A. pereshkhoranicus* Maassoumi & F.Ghahrem., and *A. straussii* Hausskn. ex Bornm., to represent the boundaries of the section. Furthermore, two species from adjacent sections (*A. campylanthus* Boiss. from sect. *Campylanthus* Bunge, and *A. cephalanthus* DC. from sect. *Microphysa* Bunge) and one outgroup species (*Oxytropis kotschyana* Boiss. & Hohen.) were included for comparison. All ITS and *ycf*1 sequences for these datasets were obtained from our previous study (Bagheri et al. 2017), with the exception of the newly identified species. As *ycf*1 sequences for this species were not available in the previous study, we conducted sequencing for this gene (DNA extraction, PCR amplification, and primers as described in Bagheri et al. 2017) and submitted the data to GenBank. Detailed information regarding the samples and their GenBank accession numbers is provided in Table S1. In summary, a combined ITS + *ycf*1 dataset with a total length of 1580 bp was generated and analyzed using Bayesian Inference (BI) in MrBayes 3.2 (Ronquist et al. 2012). The best-fit model for the analysis, selected by AIC in MrModeltest 2.3 (Nylander 2004), was HKY + Γ. In the BI analysis, two runs of four chains each were conducted for 2 million generations, with the first 25% of trees from each run discarded as burn-in. The resulting phylogenetic tree was visualized with FigTree v1.4.2 (http://tree.bio.ed.ac.uk/software/figtree/) and is presented in Fig. 3.

### Genome size and chromosome counting

This analysis was based on our previous comprehensive study on genome size and ploidy levels in the *Hymenostegis* section (Bagheri et al. 2022). The genome size of *A. chehreganii* was reported in that study, and here we estimated the genome sizes of the other two species in this complex, *A. hakkianus* and the newly identified taxon, following the protocol outlined in Bagheri et al. (2022). Genome size was estimated for three individuals from each of the two species. The genome size estimates for these two species were then compared with that of *A. chehreganii*, with detailed information presented in Table 2. Chromosome counting for the newly identified species was also performed following the protocol detailed in Bagheri et al. (2022). Unfortunately, due to the lack of successful germination, chromosome counting for the other two species in this group (*A. chehreganii* and *A. hakkianus*) was not possible. However, based on the established correlation between genome size, ploidy levels, and chromosome numbers within this section, we can infer the chromosome numbers for these species, which will be discussed in detail in the following sections.

## Results

### Morphological analysis

The morphological analysis of *Astragalus kuzehrashensis* identified several key distinguishing features in comparison to *A. chehreganii* and *A. hakkianus*. The most significant differences were observed in stipule size and texture, leaflet shape and indumentum, peduncle length and indumentum, inflorescence shape, and calyx indumentum. These morphological characteristics, which are essential for detailed species identification, are summarized in Table 1.

**Table 1:** Comparative morphological characteristics of *Astragalus Chehreganii*, *A. Hakkianus*, and *A. kuzehrashensis*

Characteristic	A. chehreganii	A. hakkianus	A. kuzehrashensis
Stipule	Thinly membranous	Chartaceous	Hyaline at the apex, thicker toward the base
Stipule length	6–10 mm	10–14 mm	16–23 mm
Petiole length	1–3 mm	2–6 mm	1.5–5 mm
Rachis indumentum	Appressed to subappressed hairs	Ascending hairs	Appressed to subappressed hairs
Leaflet shape	Narrowly elliptic	Elliptic-obovate	Narrowly elliptic
Leaflet indumentum	Appressed to subappressed hairs	Subappressed to ascending hairs	Appressed to subappressed hairs
Peduncle length	9–22 cm	7–13 cm	13–20 cm
Peduncle indumentum	Sparser and longer at the top, appressed-subappressed hairs	Mixed indumentum, tangled and ascending hairs	Sparsely hairy at the top, longer at the base, appressed-subappressed hairs
Peduncle hair length	0.1–0.5 mm	0.5–1.5 mm (up to 3.5 mm)	0.6–1.5 mm
Bract indumentum	Glabrous or sparse, occasionally at the base	Sparse at the apex, ascending hairs	Densely hairy at the apex, appressed hairs
Inflorescence shape	Globose	Globose to elliptic	Elliptic to ovoid
Inflorescence length	3–3.5 cm	3–5 cm	3.5–6 cm
Calyx indumentum	Densely hairy	Short tangled hairs mixed with long ascending hairs	Densely hairy, appressed-subappressed hairs
Standard length	16–19 mm	14–17 mm	17–19 mm

### Molecular phylogenetics

A molecular phylogenetic tree was constructed based on ITS and *ycf*1 sequences to determine the genetic relationships between the newly identified species (*A. kuzehrashensis*) and its close relatives (*A. chehreganii* and *A. hakkianus*) (Fig. 3). The phylogenetic analysis revealed that *A. kuzehrashensis* forms a clade with *A. chehreganii* and *A. hakkianus*, but the genetic differences among these species are small. The *ycf*1 sequences of these three species were found to be identical, showing no genetic divergence in this region. However, slight variations were observed in the ITS sequences. Specifically, two nucleotide differences were identified at positions 411 and 551. At position 411, *A. kuzehrashensis* has a G, while *A. chehreganii* and *A. hakkianus* share an A. At position 551, both *A. hakkianus* and *A. kuzehrashensis* have a T, whereas *A. chehreganii* has an A. These minor genetic differences indicate that while these species are closely related, they exhibit slight sequence divergence in the ITS region. This suggests that the three species are genetically similar, with *A. kuzehrashensis* showing only minimal divergence from *A. chehreganii* and *A. hakkianus*.

### Seed micromorphology

**Fig. 4:**
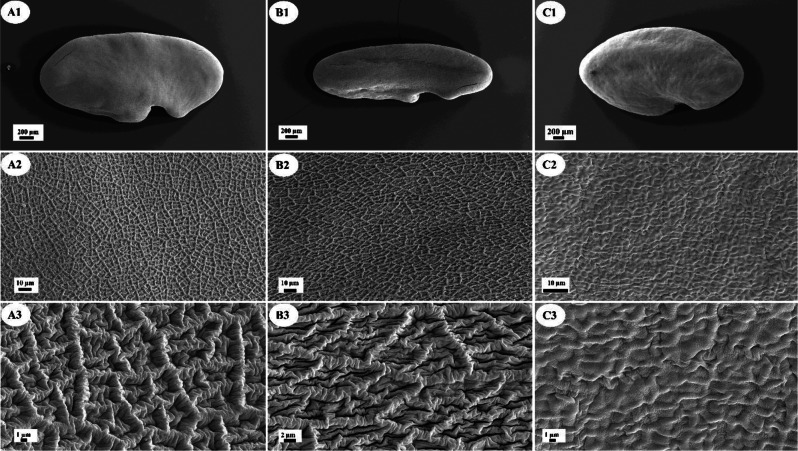
Comparative SEM images of seed micromorphology in three species within the studied complex (**A1**-**A3** *Astragalus chehreganii*; **B1**-**B3** *A. hakkianus*; **C1**-**C3** *A. kuzehrashensis*) Scale bars for each image are indicated within the figure

The seed micromorphology and shape of the three species studied, *A. chehreganii* (A), *A. hakkianus* (B), and *A. kuzehrashensis* (C) (Fig. 4), were analyzed using SEM. The seeds of *A. chehreganii* displayed a reticulate-rugulate surface ornamentation and an ovoid shape, characterized by a smooth and symmetrical appearance with well-defined, network-like patterns. In *A. hakkianus*, the seeds exhibited a more complex double reticulate-rugulate pattern and a mitiform shape, with a dual network structure on the surface, giving the seeds a slightly more pointed and intricate form. In contrast, the seeds of *A. kuzehrashensis* showed a distinctly different rugulate ornamentation, combined with a reniform (kidney-shaped) structure, marked by irregular, deeply folded elevations and a highly undulating surface. These morphological differences in seed surface ornamentation and shape underscore the diversity within these species and suggest that these characters could be significant in distinguishing species within the *Hymenostegis* section. The SEM images illustrating these features are presented in Fig. 4.

### Genome size and chromosome numbers

Genome size estimation was conducted for the newly identified species *A. kuzehrashensis*, as well as for *A. chehreganii* and *A. hakkianus*. The genome size of *A. chehreganii* was previously measured and reported as 2C = 4.61 pg (Bagheri et al. 2022). In this study, the genome size for two individuals of *A. hakkianus* was measured, with an average value of 2C = 7.42 pg. Additionally, for *A. kuzehrashensis*, the genome size was determined to be an average of 2C = 7.00 pg across two individuals. These measurements suggest that *A. hakkianus* and *A. kuzehrashensis* possess larger genomes compared to *A. chehreganii*. Additional detailed information on genome size measurements is provided in Table 2.

**Table 2:** Detailed genome size measurements for *Astragalus chehreganii*, *A. hakkianus*, and *A. kuzehrashensis*, including mean values, coefficients of variation (CV%), and standard references used. Asterisk: data taken from Bagheri et al. (2022)

Species	Genome size standard	Mean standard	Mean sample	CV% standard	CV% sample	2C genome size (pg)
A. chehreganii*	*Zea mays*	100.12	85	2.86	4.57	4.61
*A. hakkianus*	*Zea mays*	105.28	143.82	3.4	4.48	7.42
*A. kuzehrashensis*	*Zea mays*	100.64	129.66	3.72	5.33	7.00

**Fig. 5:**
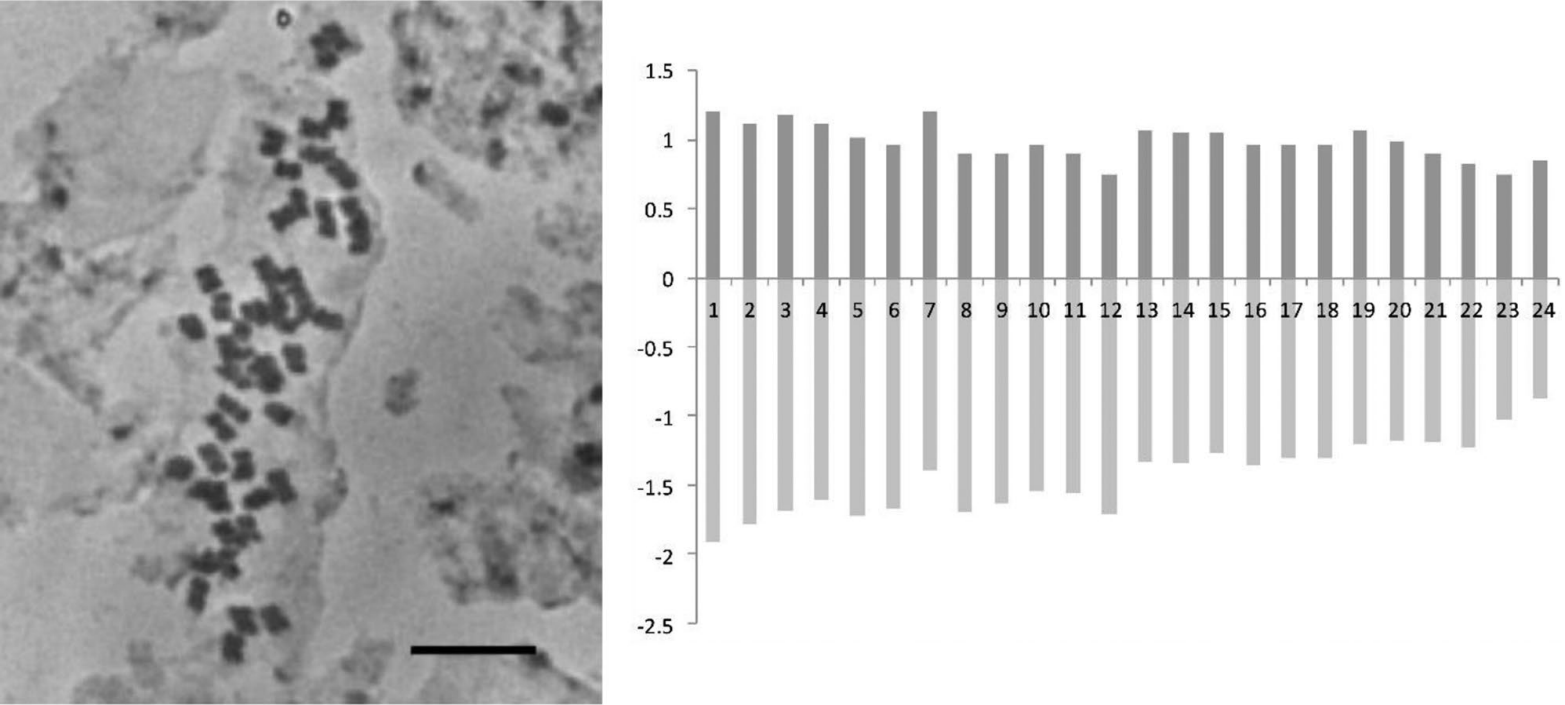
Mitotic metaphase chromosomes and idiogram of *Astragalus kuzehrashensis*, illustrating the chromosome number (2*n* = 6*x* = 48). Scale bar = 10 μm

Chromosome counting for *Astragalus kuzehrashensis* revealed a chromosome number of 2*n* = 6*x* = 48, indicating a hexaploid level. Due to unsuccessful germination, chromosome counts for *A. chehreganii* and *A. hakkianus* were not obtained. For *A. kuzehrashensis*, detailed karyotypic analysis showed a karyotypic formula (KF) of 1 M + 17 m + 6sm, with a mean chromosome length (CL) of 2.42 μm. The arm ratios (p/q) were 1.43 and 0.98, respectively, with a centromeric index (Ci) of 0.42. Additional metrics include a total form percentage (TF%) of 41%, symmetric index (Syi%) of 68%, and an asymmetry index (Ask%) of 59%. The mitotic metaphase chromosomes and idiogram for *A. kuzehrashensis* are illustrated in Fig. 5.

### Taxonomy

***Astragalus kuzehrashensis*** Karbalaei, Bagheri & Maassoumi, sp. nov. Figure 2A–G.

#### Diagnosis

Differt ab *Astragalus chehreganii* Podlech & Zarre stipulis hyalinis apice, crassioribus ad basin (nec tenuiter membranaceis), 16–23 mm (nec 6–10 mm) longis; petiolis 1.5–5 mm (nec 1–3 mm) longis; foliolis anguste ellipticis, utrinque pilis appressis vel subappressis, racemis ellipticis ad ovoideis 3.5–6 cm longis (nec globosis, 3–3.5 cm); bracteis apice dense pilosis (nec glabris vel sparse pilosis); calycibus dense pilis appressis-subappressis vestitis (nec dense pilosis). Ab *Astragalus hakkianus* Bagheri, Maassoumi & Rahimin. differt stipulis hyalinis apice, crassioribus ad basin (nec chartaceis), 16–23 mm (nec 10–14 mm) longis; foliolis anguste ellipticis, utrinque pilis appressis vel subappressis (nec elliptico-obovatis); pedunculis 13–20 cm (nec 7–13 cm) longis; racemis ellipticis ad ovoideis 3.5–6 cm longis (nec globosis ad ellipticis, 3–5 cm); calycibus dense pilis appressis-subappressis vestitis (nec pilis brevioribus confertis pilis longioribus mixtis); vexillo 17–19 mm (nec 14–17 mm) longo.

#### Description

Plants subshrubby, caespitose, spiny, 20–30 cm tall. Stems 10–16 cm long (rarely shorter), with older parts woody, densely covered with remnants of last year’s petioles, and branched; stipules up to 23 mm long, thickly membranous, hyaline at the apex, sometimes becoming chartaceous toward the base, adnate to the petiole, connate, glabrous, margins ciliate. Leaves 2.5–12 cm long; petiole 1.5–5 cm long, rachis densely covered with appressed to subappressed hairs 0.3–1 mm long, spiny. Leaflets in 7–9 pairs, narrowly elliptic, 5–17 × 1.5–3 mm, acuminate, pungent, with a cusp 0.5–3 mm long, on both sides densely covered with appressed hairs 0.5–1 mm long. Peduncles 13–20 cm long, longer than the leaves, densely covered with appressed to subappressed hairs 0.1–0.6 mm long, sometimes mixed with long ascending hairs up to 1.5 mm long. Racemes elliptic to ovoid, 4–6.5 cm long, 3–3.5 cm wide, densely many-flowered. Bracts glumaceous to thickly membranous, hyaline at the margins and apex, ovate, 12–16 × 3–6 mm, with a long acuminate tip up to 5 mm, glabrous, but ciliate at the base and margins with hairs up to 0.5 mm long. Calyx 13–17 mm long, pale yellowish, with purplish veins on the teeth, densely covered with short, tangled, appressed hairs 0.3–0.5 mm mixed with longer ascending hairs 1.5–2.5 mm long; teeth subulate, purple, 5–8 mm long. Petals with violet limbs and pale yellow claws; standard 17–19 mm long, blade oblong-panduriform, 5–8 mm wide, slightly emarginate, constricted at the middle, narrowly hastate at the base, gradually narrowing into a short claw. Wings 15–19 mm long, blades narrowly elliptic, obtuse, 2.5–3.5 mm wide; auricle 0.5 mm, claw 7–10 mm. Keel 14–16 mm long, blades obliquely obovate, 5 × 3 mm, with a rounded apex, minute auricle, and claw 9–10 mm long. Ovary sessile, white hairy, 7 × 3 mm. Fruit a legume, densely hairy like the ovary, seed solitary, brown, reniform, ca. 3 × 1.6 mm, with a relatively pitted surface.

#### Type

Iran. West Azarbaijan province: From Serow to Salmas, ca. 30 km before Salmas, towards Kuzehrash, 38° 01’ 47"N, 44° 48’ 26"E, 1700 m, 28 June 2014, Bagheri 101,436 (holotype TARI!, isotype HUI!, MSB! ).

#### Additional specimens examined (paratypes)

Same locality, 38° 01’ 61"N, 44° 48’ 13"E, 1810 m, 20 July 2022, Bagheri 25827, HUI!; Same locality, 38° 01’ 54"N, 44° 48’ 19"E, 1800 m, 10 August 2019, Bagheri 25828, HUI!; West Azarbaijan province: From Salmas to Kuzehrash, 38° 01’ 47"N, 44° 48’ 26"E, 1700 m, 20 July 2013, Bagheri 98,062, TARI!, HIU!

#### Etymology

The species is named *Astragalus kuzehrashensis* in reference to the village of Kuzehrash, located in Salmas County, West Azarbaijan province, Iran. This naming highlights the geographical origin of the species and honors the local biodiversity of this region.

#### Distribution and habitat

*Astragalus kuzehrashensis* is currently known from two small populations located at its type locality in West Azarbaijan province, northwestern Iran, near Kuzehrash (Fig. 1). The species is a distinctive element of the Irano-Turanian floristic region, thriving on rough, rocky mountain slopes at elevations between 1700 and 1810 m. *Astragalus kuzehrashensis* forms dense, spiny thorn cushion shrubs on steep slopes, a characteristic vegetation type within this floristic region. Flowering occurs from June to July, with fruiting shortly after, and seeds are produced by August. Figure 2 illustrates the typical habitat, growth form, and detailed floral characteristics of the species.

#### Conservation status

**Fig. 6:**
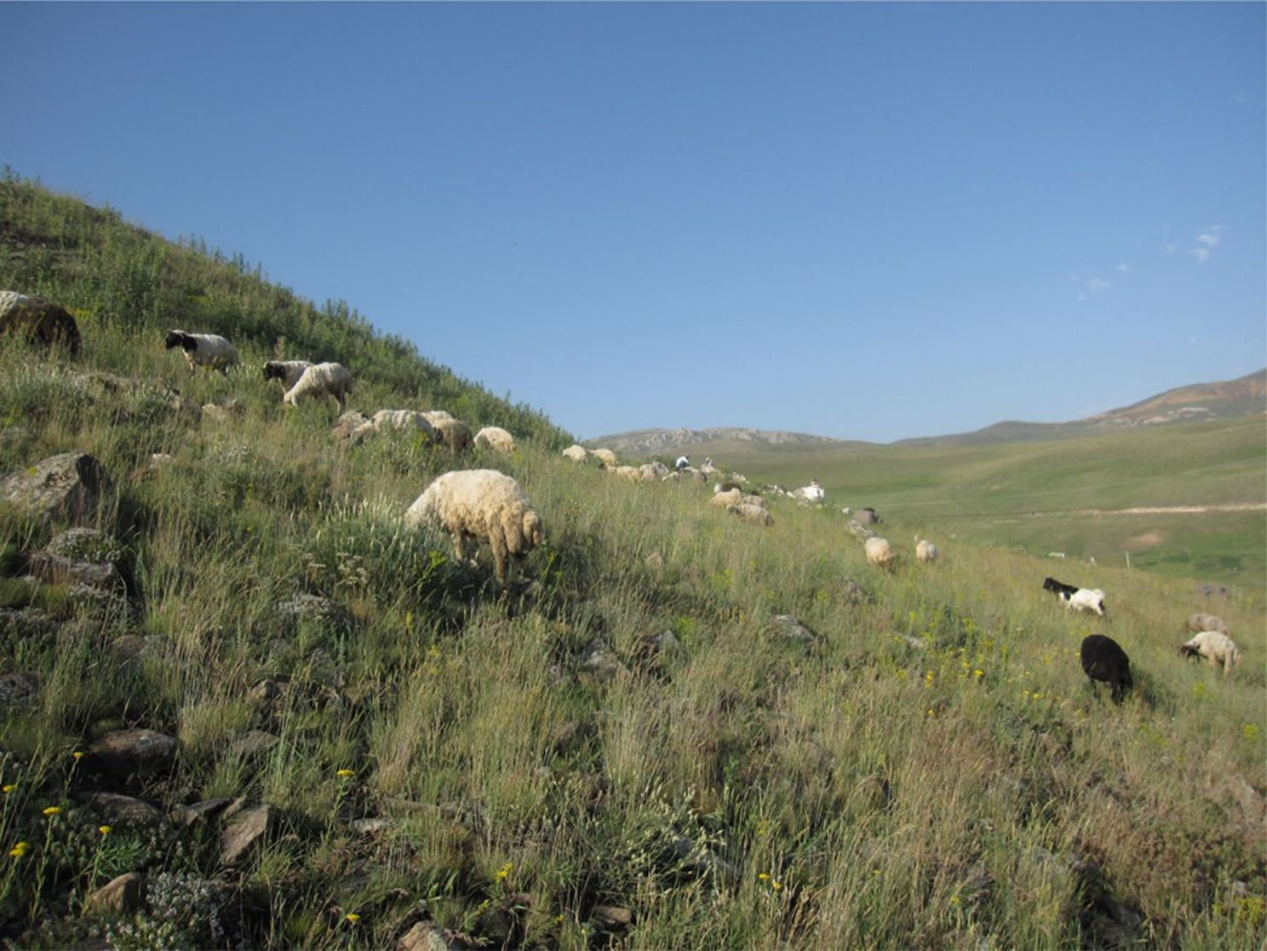
Grazing livestock in the habitat of *Astragalus kuzehrashensis*, illustrating the pressure on its habitats

The distribution of *A. kuzehrashensis*, restricted to a specific area in northwestern Iran, increases significant concerns regarding its conservation. The species inhabits rocky slopes within the region, areas increasingly vulnerable to environmental changes and human activities. One of the most immediate threats to *A. kuzehrashensis* is overgrazing, as evident in field observations (Fig. 6). The continuous grazing pressure from livestock in this region stances a considerable risk to the species’ survival, potentially leading to habitat degradation and a reduction in population size. Given its limited geographic range and the mounting pressures on its habitat, *A. kuzehrashensis* warrants attention in conservation planning.

## Discussion

This study focuses on the newly identified *Astragalus kuzehrashensis* and its differentiation from its closest relatives within the section *Hymenostegis*, *A. chehreganii* and *A. hakkianus*. Although our comparisons primarily involved these two species, it is important to note that the section *Hymenostegis* comprises over 80 species, many of which are found in West Azarbaijan province, including several local endemics and species extending into eastern Turkey, such as *A. pereshkhoranicus* Maassoumi & F.Ghahrem., *A. bradosticus* Maassoumi & Podlech, *A. zohrabi* Bunge, *A. ciloensis* Podlech, and others (Maassoumi and Ghahremaninejad 1999; Podlech and Zarre 2013; Maassoumi 2020). This highlights the significance of northwestern Iran, particularly the West Azarbaijan province, as a key biodiversity hotspot for section *Hymenostegis*. Despite the wide diversity within *Hymenostegis*, the significant morphological differences between the species in our study complex and other members of the section justify our focused comparison on *A. chehreganii* and *A. hakkianus*.

*Astragalus kuzehrashensis* shows several key morphological differences from its close relatives, *A. chehreganii* and *A. hakkianus*, as detailed in the morphological analysis. These differences are crucial for species identification within the genus *Astragalus*, particularly within *Hymenostegis*. The seed micromorphology analysis, which revealed distinct surface ornamentation patterns in *A. kuzehrashensis*, further supports its taxonomic distinction from closely related taxa. The morphological differences of *A. kuzehrashensis* in contrast to high molecular similarity, suggesting a process of recent divergence where morphological differentiation has occurred relatively rapidly, despite close genetic relationships (Bagheri et al. 2017).

The molecular phylogenetic analysis, based on ITS and *ycf*1 sequences, indicates that *A. kuzehrashensis* forms a clade with *A. chehreganii* and *A. hakkianus*. However, the genetic divergence within this clade is minimal, particularly as *ycf*1 sequences were identical among the species. The slight variations observed in the ITS sequences, provide some evidence for the distinct status of *A. kuzehrashensis* within this species complex. This minimal genetic divergence, coupled with the notable morphological differences, emphasizes the importance of an integrative taxonomic approach. Combining molecular data with detailed morphological and micromorphological observations is essential for accurate species delimitation within complex groups like *Hymenostegis*.

The integration of genome size estimation and chromosome counting further strengthens the taxonomic framework for *A. kuzehrashensis*. Our study found that the genome size of *A. kuzehrashensis* (2 C = 7.00 pg) is consistent with its hexaploid chromosome count (2n = 6x = 48). The genome size of *A. hakkianus* (2 C = 7.42 pg) suggests it is also likely a hexaploid, whereas *A. chehreganii* (2 C = 4.61 pg) is a tetraploid species (Bagheri et al. 2022). This correlation between genome size, ploidy levels, and chromosome numbers within the *Hymenostegis* section suggests a direct relationship that can be used to infer ploidy levels and chromosomal configurations in closely related species. These findings indicate that *A. kuzehrashensis* and *A. hakkianus* share a similar ploidy level, which may have facilitated their morphological divergence from *A. chehreganii*.

## Conclusion

*Astragalus kuzehrashensis* has been identified and described as a new species within the genus *Astragalus* through a comprehensive approach that integrates morphological, molecular, micromorphological, and cytogenetic analyses. The results of this study not only establish the distinctiveness of *A. kuzehrashensis* but also highlight the value of employing multiple lines of evidence in the taxonomic revision of complex groups like the section *Hymenostegis*. Moreover, this research highlights the importance of ongoing exploration and conservation efforts, particularly in the Irano-Turanian region, where many species may remain cryptic or poorly understood due to their recent evolutionary divergence.

## Electronic supplementary material

Below is the link to the electronic supplementary material.

Supplementary Material 1

## Data Availability

The herbarium specimens were deposited in the above-mentioned herbaria. DNA sequences were uploaded to the NCBI nucleotide database and are freely available.
