# Supplementary material for: A new species of Astragalus (Fabaceae) from the Irano-Turanian biodiversity hotspot: an integrative approach
Source: Bot Stud. 2025 Jan 8;66:1. doi: 10.1186/s40529-024-00448-6 (PMC11711607; doi:10.1186/s40529-024-00448-6)
Supplement: Supplementary file 1 — Supplementary Material 1 [file 40529_2024_448_MOESM1_ESM.docx]

**Table S1** Details of the samples used in the phylogenetic analysis, including accession numbers from GenBank.

| **Species** | **Voucher** | **ITS accession number** | **ycf1 accession number** |
| --- | --- | --- | --- |
| *Astragalus chehreganii* | 41877, MSB, Isotype | LT622401 | KY071141 |
| *Astragalus chehreganii* | 98410, TARI | LT622402 | - |
| *Astragalus hakkianus* | 98057, TARI, Holotype | LT622444 | KY071152 |
| *Astragalus hakkianus* | 98059, HUI | LT622445 | - |
| *Astragalus kuzehrashensis* | 101436, TARI, Holotype | LT622463 | PQ594183 |
| *Astragalus kuzehrashensis* | 98062, HUI | LT622464 | PQ594184 |
| *Astragalus hymenostegis* | TARI, 98072 | LT622456 | KY071155 |
| *Astragalus straussii* | TARI, 97917 | LT622606 | KY071185 |
| *Astragalus cephalanthus* | TARI, 63774 | LT622398 | KY071139 |
| *Astragalus campylanthus* | TARI, 17497 | LT622400 | KY071140 |
| *Oxytropis kotschyana* | TARI, 98085 | LT622640 | - |
